# The differential effect of the free maternity services policy in Kenya

**DOI:** 10.4102/phcfm.v11i1.1887

**Published:** 2019-05-27

**Authors:** Henry O. Owuor, Stephen A. Asito, Samson O. Adoka

**Affiliations:** 1 Department of Family Medicine, School of Medicine, Moi University, Kesses, Uasin Gishu County, Kenya; 2 Department of Biological Sciences, Jaramogi Oginga Odinga University of Science and Technology, Bondo, Kenya; 3 Department of Public Health, School of Health Sciences, Jaramogi Oginga Odinga University of Science and Technology, Bondo, Kenya

**Keywords:** free maternity services policy, deliveries attended by skilled birth attendants, skilled care delivery, facility type, sub-county, Nyamira County, Kenya region

## Abstract

**Background:**

The Government of Kenya introduced the free maternity services (FMS) policy to enable mothers deliver at a health facility and thus improve maternal health indicators.

**Aim:**

The aim of this study was to determine if there was a differential effect of the policy by region (sub-county) and by facility type (hospitals vs. primary healthcare facilities [PHCFs]).

**Setting:**

The study was conducted in Nyamira County in western Kenya.

**Methods:**

This was an interrupted time series study where 42 data sets (24 pre- and 18 post-intervention) were collected for each observation. Monthly data were abstracted from the District Health Information System-2, verified, keyed into and analysed by using IBM-Statistical Package for the Social Sciences (SPSS-17).

**Results:**

The relative effect of the policy on facility deliveries in the county was an increase of 22.5%, significant up to the 12th month (*p* < 0.05). The effect of the policy on deliveries by region was highest in Nyamira North and Masaba North (*p* < 0.001 up to the 18th month). The effect was larger (46.5% vs. 18.3%) and lasted longer (18 months vs. 6 months) in the hospitals than in the PHCFs. The increase in hospital deliveries was most significant in Nyamira North (61%; *p* < 0.001). There was a medium-term effect on hospital deliveries in Borabu (up to 9 months) and an effect that started in the sixth month in Manga. The relative effect of the policy on facility deliveries in PHCFs was only significant in Nyamira North and Masaba North (*p* < 0.001).

**Conclusion:**

The effect of the FMS policy was varied by region (sub-county) and by facility type.

## Introduction

Kenya has not made sufficient progress towards improving maternal health because there is a high maternal mortality rate (MMR) and low utilisation of maternal health services.^1^ At 362 maternal deaths per 100 000 live births, the MMR for Kenya is more than double the global average and well above the Millennium Development Goal (MDG) target of 147/100 000 live births by 2015.^1,2,3^ It is only 61% of deliveries that are conducted under the supervision of a skilled birth attendant.^3^ Moreover, more than half (52%) of women do not attend four antenatal care (ANC) visits.^4,5^ This is attributed to poverty and distance from home location to health facilities, which have been identified as the main barriers to having skilled attendance at childbirth.^6,7,8,9^ In Nyamira County, the MMR, proportion of women attending fourth ANC and having skilled attendance at birth were 385/100 000 live births, 56% and 41%, respectively.^10,11^

Because of the insufficient progress towards improving maternal health, the Government of Kenya (GoK) introduced the Free Maternity Services (FMS) policy to enable mothers to deliver at a health facility nearest to them at no cost at all on 01 June 2013. The policy is financed by the National Government. Under the programme, the health centres and dispensaries, that is, primary healthcare facilities (PHCFs), are reimbursed Ksh2500.00 ($27.70) for every delivery, whereas the hospitals are reimbursed KSh5000.00 ($55.60) for every delivery, normal or caesarean. These funds are paid directly to the facilities.

Generally, removal of user fees often results in increases in the use of health services, as has been the case in South Africa and Mali.^12,13^ Meanwhile, it has been demonstrated that differences in uptake of maternal health services may be associated with context-specific factors that may modify the effect of a policy.^9^ At the service level, it may be because of the availability and physical accessibility of these services^14,15,16,17,18^; at community level, these factors include a lack of basic health knowledge among expectant mothers, financial barriers to access and perceived low quality of services,^15,16,17,18,19^ whereas at the individual level residence (whether urban or rural), health beliefs and personal characteristics of the users may be the effect modifiers.^20,21,22,23^

A baseline report by the Ministry of Health (MoH) demonstrated that only 41% of all deliveries in public health facilities occur in PHCF despite the geographical and financial accessibility.^24^ The report also states that hospital-level care is out of reach for many people because of distance, which necessitates additional transportation costs and the cost of the service provided.^24^ There is paucity of studies that have compared the effect of a similar policy by facility type and by region.

Therefore, this interrupted time series (ITS) study was undertaken to determine if there was a differential effect of the FMS policy by region (sub-counties) and by facility type (hospitals vs. PHCFs, that is, dispensaries and health centres) in the sub-counties in Nyamira County.

## Research methods and designs

### Study design

The ITS study design was used to conduct the study. This is a quasi-experimental longitudinal study design that involves statistical comparison of time trends before and after an intervention. It was used because there was a definite point in time when the implementation of the policy began and data could be obtained from the District Health Information System (DHIS-2) for the time period before and after policy implementation. There was no parallel event that would have affected utilisation of maternal health services for deliveries supervised by skilled birth attendants in the county. Devolution of health services was delayed and mainly focused on the provision of services. However, the study design and the method of analysis would remove the effect of any confounding factors or biases that may affect the results of the study. The manner in which deliveries from health facilities were captured and reported to the DHIS-2 did not change in any way to affect study findings (i.e. there was no change in mode of measurement).

### Setting

The study was conducted in Nyamira County, one of the 47 counties in Kenya. Nyamira County is in the former Nyanza province and borders Homabay County to the north, Kisii County to the west, Bomet County to the south-east and Kericho County to the east. Nyamira County was selected for the study because of its maternal health indicators, particularly uptake of maternal health services and maternal mortality. The county covers an area of 899.4 km^2^ and is home to five sub-counties: Nyamira (South), Nyamira North, Borabu, Manga and Masaba North.^25^ The county had a population of 598 252 persons in the 2009 housing and population census report but was projected to cross 667 716 persons in 2015.^4^

There are 130 health facilities in the county and eight of these are hospitals. The rest are dispensaries and health centres (i.e. PHCF). The average distance to a health facility in Nyamira County is 7 km and agriculture is the main economic activity, with tea and coffee being the main cash crops grown.^10^

### Study population and sampling strategy

All the deliveries from all the health facilities were incorporated so as to achieve population-level outputs and outcomes and representativeness. Data were therefore collected for all the health facilities in the county.

### Data collection

Data were abstracted from the DHIS-2 for each sub-county, and for all the hospitals and the PHCFs in the county using a data abstraction form. These were then verified using facility-level data. Data were collected for the period between 01 June 2011 and 30 November 2014. A records officer was trained and employed to collect the data from the DHIS-2 and to enter the findings in an Excel spreadsheet. Data from hospitals and the PHCFs were summated to find the total from hospitals and PHCFs, respectively. Data were also retrieved by sub-county. The retrieved data were verified by a second officer who counter-checked if the entries were similar with what was in the DHIS-2 and with facility-level data.

### Data analysis

After data verification and cleaning, the data were transferred to the International Business Machines Corporation-Statistical Package for the Social Sciences (IBM-SPSS) version 17 for an ITS analysis using the auto-regressive integrated moving-average (ARIMA) model that takes into account any time or cyclical trends and autocorrelation among observations so that there are no overestimations or underestimations of the intervention effects. Analysis was performed for each sub-county, and by facility type (hospital and PHCF) in the sub-county.

The regression model used for the study given the coefficient *β*1 is for time, *β*2 for phase and *β*3 for interact was:

Births attended by skilled attendant [skilled care deliver (SCD)] = *β*0 (constant) + *β*1 (time) + *β*2 (phase) + *β*3 (interact),

Where,

*β*0 estimates the baseline SCD at the beginning of the pre-FMS period.

*β*1 estimates the change in number of SCD that occurs with each month before the FMS policy.

*β*2 estimates the change in SCD immediately after the FMS policy.

*β*3 estimates the change in the trend of SCD of the post-FMS period compared to the pre-FMS period.

Estimates for regression coefficients corresponding to two standardised effect sizes are obtained: a change in level (step change, i.e. *β*2) and a change in trend before the intervention (*β*1). The change in trend after the intervention (*β*3) is the sum of the pre-intervention slope and the change in level, that is, *β*1 + *β*2. Other coefficients generated from the ‘ARIMA model parameters’ including the corresponding standard error and the *t*-values were important in calculating the 95% confidence intervals (CI). The *p*-values demonstrated the significance of the effect of the free maternity services policy by sub-county, by hospital and by PHCF per sub-county. Percentages were calculated to estimate the relative effect of the policy in the various sub-counties and by health facility type. This facilitates comparison between the sub-counties and health facilities. The analysis was performed to determine the first- and third-month-level effect after the policy execution date to monitor any immediate and short-term effects of the policy. Analysis for the sixth and ninth months was to demonstrate any mid-term effects, whereas the analyses performed at the 12th- and 18th-month post-intervention were to assess whether the policy had a long-term effect. Statistical significance was set at *p* ≤ 0.05.

### Ethical considerations

This study was approved by Jaramogi Oginga Odinga Teaching and Referral Hospital’s Ethics Review Committee (accreditation number 01713). As this study involved data abstraction, no written consent was obtained from the study participants for using their records; however, participant reports or information was anonymised and de-identified prior to analysis.

## Results

There were 130 facilities and five sub-counties reporting to the DHIS-2 in Nyamira County.

Table 1 demonstrates that over a 24-month period before the FMS policy implementation, 20 840 deliveries were reported, whereas over the 18 months after the policy implementation 23 570 deliveries were reported. This represents a 51% increase in the average facility deliveries per month. There was also a 25% increase in the average fourth ANC visits per month over the two periods. Eight new PHCFs became operational within the county during the post-intervention. There was also a marked reduction in the birth-related complications reported (73.8%).

**TABLE 1:** Population and service data for Nyamira County before and after the free maternity services policy and deliveries attended by skilled care providers by sub-county.

Variable	Pre-intervention	Post-intervention
Population data	June 2011–May 2013	June 2013–November 2014
County Pop	637 295	661 106
Expected deliveries	21 668	22 478
Average expected deliveries per month	902.8	1248.8
Number of hospitals	8	8
Number of PHCFs	114	122
Total number of health facilities	122	130
Number of sub-county	5	5
Service data	-	-
Hospital deliveries	7972	8667
PHCF deliveries	12 868	14 903
Total facility-based deliveries and (ratio)†	20 840 (96.2%)	23 570 (104.9%)
Average facility deliveries per month	868.3	1309.4
Fourth ANC visits and (ratio)‡	14 947 (69.0)	14 051 (62.5%)
Average fourth ANC per month	622.8	780.6
Caesarean sections performed and (ratio)§	753(3.6%)	809(3.4%)
Average CS per month	31.4	44.9
Maternal deaths	21	18
Complications	84	22
Neonatal deaths	141	142
SCD by sub-counties	-	-
Borabu	3250	3224
Manga	1986	2171
Masaba North	3775	4813
Nyamira South	9148	9326
Nyamira North	3762	4886

PHCF, primary healthcare facility; ANC, antenatal care; SCD, skilled care deliveries; CS, caesarean section.

† , Total facility deliveries × 100 / expected deliveries.

‡ , Fourth ANC × 100 / expected deliveries.

§ , Caesarean sections performed × 100 / total facility-based deliveries.

There was an increase in facility deliveries for all sub-counties. Borabu, Manga, Masaba North, Nyamira South, and Nyamira North had 32.6%, 45.8%, 70.0%, 40.0% and 72.6% increases in facility deliveries per month, respectively.

There had been an increase of ten facility deliveries per month (95% CI: 3.4–16.8; *p* = 0.004) in the county before the FMS policy. Following the policy implementation, there is a significant increase in the number of deliveries up to the 12th month, with the relative effect of the policy on this increase in facility deliveries oscillating between 21% and 25%. Looking at relative effects (RE of the policy in the above tables (tables 2 and 3), we note that the policy had its largest effect in Nyamira North and Masaba North sub-counties, with the least effects realised in Nyamira South and Borabu sub-counties. In fact, the effect of the FMS policy was only significant in the long-term in Nyamira North and Masaba North. There was no significant effect in the other sub-counties except in Borabu where there was some significant immediate effect at 1-month post-policy implementation (*p* = 0.046).

**TABLE 2:** Parameter estimates for Nyamira South and Nyamira North and by facility type for free maternity services policy from June 2011 to November 2014.

Variable	Nyamira County			Nyamira South			Nyamira North		
	LE	*p*	RE (%)	LE	*p*	RE (%)	LE	*p*	RE (%)
**SCD**									
Pre-slope	10.086	0.004	-	5.836	0.003	-	0.510	0.385	-
Interact	−1.895	0.753	-	−4.563	0.198	-	1.027	0.338	-
Post-slope	8.191	-	-	1.273	-	-	1.537	-	-
1	257	< 0.001	24	53	0.136	12	95	0.000	57
3	254	< 0.001	25	48	0.157	10	97	0.000	59
6	249	0.001	23	30	0.415	6	100	0.000	60
9	244	0.003	23	16	0.694	4	103	0.000	62
12	239	0.009	21	3	0.956	1	106	0.000	62
18	228	0.055	19	−25	0.702	−4	112	0.000	65
**SCD at hospitals**									
Pre-slope	−0.390	0.833	-	−0.466	0.563	-	−0.130	0.689	-
Interact	5.458	0.114	-	2.265	0.131	-	1.979	0.002	-
Post-slope	5.068	-	-	1.799	-	-	1.849	-	-
1	116	0.004	34	34	0.050	24	42	0.000	79
3	127	0.001	38	37	0.025	27	46	0.000	78
6	143	0.001	42	45	0.011	32	52	0.000	63
9	160	0.001	49	52	0.008	38	57	0.000	91
12	176	0.001	53	59	0.008	43	63	0.000	121
18	209	0.002	63	72	0.012	56	15	0.000	153
**SCD at PHCF**									
Pre-slope	8.967	0.002	-	6.154	0.000	-	0.546	0.191	-
Interact	−9.224	0.070	-	−7.388	0.018	-	−0.748	0.325	-
Post-slope	−0.257	-	-	−1.234	-	-	−0.202	-	-
1	189	0.001	29	32	0.249	10	55	0.000	54
3	185	0.001	28	25	0.357	8	53	0.000	52
6	143	0.012	20	−5	0.867	−1	51	0.000	49
9	116	0.062	17	−27	0.431	−8	49	0.000	46
12	88	0.210	12	−59	0.228	−13	46	0.000	43
18	33	0.723	4	−94	0.096	−22	42	0.005	38

FMS, free maternity services; LE, level effect; RE, relative effect; SCD, skilled care deliveries; PHCF, primary healthcare facility.

**TABLE 3:** Parameter estimates for Borabu, Manga and Masaba North Sub-county by facility type for free maternity services policy (June 2011–November 2014).

Variable	Borabu			Manga			Masaba North		
	LE	*p*	RE (%)	LE	*p*	RE (%)	LE	*p*	RE (%)
**SCD**									
Pre-slope	1.468	0.184	-	0.754	0.278	-	1.142	0.051	-
Interact	−3.266	0.122	-	0.208	0.871	-	3.502	0.002	-
Post-slope	−1.798	-	-	0.962	-	-	4.644	-	-
1	41	0.047	24	19	0.172	20	56	0.000	33
3	38	0.056	24	20	0.147	20	64	0.000	37
6	25	0.251	16	20	0.161	20	74	0.000	42
9	15	0.539	10	21	0.190	21	84	0.000	46
12	5	0.854	3	22	0.235	22	95	0.000	52
18	−14	0.708	−8	23	0.338	20	116	0.000	60
**SCD at Hospitals**									
Pre-slope	0.295	0.102	-	−0.015	0.969	-	0.588	0.361	-
Interact	−0.139	0.669	-	1.077	0.131	-	0.618	0.601	-
Post-slope	0.156	-	-	1.062	-	-	1.206	-	-
1	11	0.006	64	12	0.116	43	1	0.956	1
3	11	0.005	65	14	0.063	48	3	0.837	3
6	10	0.011	56	17	0.030	58	4	0.777	4
9	9	0.025	51	21	0.021	80	6	0.701	6
12	9	0.055	46	24	0.020	103	8	0.652	8
18	8	0.176	39	30	0.025	93	11	0.607	11
**SCD at PHCF**									
Pre-slope	1.610	0.064	-	0.740	0.096	-	0.521	0.500	-
Interact	−2.914	0.074	-	−0.802	0.322	-	2.823	0.054	-
Post-slope	−1.304	-	-	−0.062	-	-	3.344	-	-
1	17	0.319	11	7	0.422	11	57	0.001	75
3	15	0.372	10	6	0.491	9	62	0.000	77
6	2	0.906	1	3	0.720	5	71	0.000	93
9	−7	0.727	−4	1	0.929	1	79	0.000	91
12	−15	0.486	−9	−2	0.895	−2	88	0.000	109
18	−33	0.286	−19	−6	0.672	−8	105	0.000	120

FMS, free maternity services; LE, level effect; RE, relative effect; SCD, skilled care deliveries; PHCF, primary healthcare facility.

The effect of the FMS policy was larger and lasted longer in the hospitals (18 months) than in the PHCFs (6 months) in the county (relative effect of 46.5% for hospitals vs. 18.3% for PHCFs). In the sub-counties, there were mixed results of the FMS policy on hospitals and PHCFs. Nyamira North had the most significant (*p* < 0.001) and long-term (up to 18 months) increase in hospital deliveries, whereas Masaba North had none. There was a medium-term effect on facility deliveries in Borabu (up to 9 months) and an effect that started later on in Manga (from the sixth month onwards).

Among the PHCFs, the effect of the FMS policy on facility deliveries was not significant in Nyamira South, Borabu and Manga, whereas it was significant in Nyamira North and Masaba North (*p* < 0.001). This varied effect of the policy by sub-county resulted in a mid-term effect of only 6 months on facility deliveries in PHCFs in the county.

## Discussion

### Key findings

Comparing the post- to the pre-intervention durations, there was a 51% and a 25% increase in the average facility deliveries and the average fourth ANC visits per month, respectively. There was an increase in facility deliveries in all the sub-counties. Borabu, Manga, Masaba North, Nyamira South and Nyamira North had 32.6%, 45.8%, 70.0%, 40.0% and 72.6% increases in facility deliveries per month, respectively. The effect of the FMS policy was larger and lasted longer in the hospitals (18 months) than in the PHCFs (6 months) in the county (relative effect of 46.5% for hospitals vs. 18.3% for PHCFs). In the sub-counties, the policy had mixed results in hospital deliveries. Among the PHCFs, the effect of the FMS policy on facility deliveries was not significant in Nyamira South, Borabu and Manga, whereas it was significant in Nyamira North and Masaba North (*p* < 0.001).

### Discussion of key findings

Despite the abolition of user fees in many countries to improve the utilisation of maternal health services, studies comparing the effect of the policy by region remain scant. This study found that there was a differential effect of the FMS policy by region (sub-counties). The FMS policy had long-term significant effects in Nyamira North and Masaba North sub-counties, but it had no significant effect in the other three neighbouring sub-counties. The observed differential effect of the policy may be influenced by the level of income, education and whether the region is urban or rural.^9^ Geographical accessibility, transportation costs and systems, availability and expertise of the health attendants and the governance and implementation capacity of the various administrators of the health departments in the sub-counties may also have an effect.^26,27,28,29^

There was also a differential effect of the FMS policy by health facility type. In the county, the effect of the policy was most marked in the hospitals than in the PHCFs. In the sub-counties, all hospitals had a significant improvement in deliveries except Masaba North. Nevertheless, only Nyamira North and Masaba North had a significant increase in deliveries at the PHCF.

In addition, there was increase in deliveries in the PHCF that was immediate but tapered off. The initial increase in utilisation of maternity services at the PHCF could be because of their proximity to the population and comparative ease of accessibility. There could have been a hesitation and reluctance to seek care at the hospitals with people fearing that the free services were only available at the PHCF. A shift by clients to seek delivery services from PHCFs to hospitals because of perceived quality of care and the availability of staff at the hospitals round the clock could have caused the sustained rise in number of deliveries attended by skilled health personnel in the hospitals. Of note is that the hospitals also receive relatively higher reimbursements per delivery than the PHCFs and may have offered better quality services, better staff rewards and better gift hampers to mothers who deliver at their facilities.

There is a dearth of studies that have compared the effect of a similar policy at the various levels of the healthcare provision system.

### Strengths and limitations

There were no comparison groups in this study and it was relatively short term. This was addressed by using a longer pre-intervention period and a rigorous analysis method. The multiple-level effects were instrumental in defining how long it took for the policy’s effect to taper off. By conducting the study as such, the results are valid and conclusions can be drawn from this study.

### Implications or recommendations

As there is paucity of studies that have compared the effect of a similar policy at the various levels of the healthcare provision system, more studies should be undertaken to assess the effect of the policy in different regions and at various levels of healthcare provision. These studies should also aim to elucidate the factors that influence a policy’s differential effect. From the findings of this study, future implementations of similar policies should be determined by the need and possible responses of the population to the policy so as to maximise effects.

## Conclusion

The effect of the FMS policy was varied by region (sub-county) and by type of health facility. The increase in deliveries in the PHCF was immediate but tapered off. However, the effect of the policy was most marked in the hospitals than in the PHCFs.
